# Treatment planning of total marrow irradiation with intensity-modulated spot-scanning proton therapy

**DOI:** 10.3389/fonc.2022.955004

**Published:** 2022-07-28

**Authors:** Darren M. Zuro, Gabriel Vidal, James Nathan Cantrell, Yong Chen, Chunhui Han, Christina Henson, Salahuddin Ahmad, Susanta Hui, Imad Ali

**Affiliations:** ^1^ Department of Radiation Oncology, University of Oklahoma Health Science Center (HSC), Oklahoma City, OK, United States; ^2^ Department of Radiation Oncology, City of Hope, Durate, CA, United States

**Keywords:** total marrow irradiation (TMI), volumetric arc radiotherapy, proton radiation therapy, dosimetric analyses, radiation therapy

## Abstract

**Purpose:**

The goal of this study is to investigate treatment planning of total marrow irradiation (TMI) using intensity-modulated spot-scanning proton therapy (IMPT). The dosimetric parameters of the intensity-modulated proton plans were evaluated and compared with the corresponding TMI plans generated with volumetric modulated arc therapy (VMAT) using photon beams.

**Methods:**

Intensity-modulated proton plans for TMI were created using the Monte Carlo dose-calculation algorithm in the Raystation 11A treatment planning system with spot-scanning proton beams from the MEVION S250i Hyperscan system. Treatment plans were generated with four isocenters placed along the longitudinal direction, each with a set of five beams for a total of 20 beams. VMAT-TMI plans were generated with the Eclipse-V15 analytical anisotropic algorithm (AAA) using a Varian Trilogy machine. Three planning target volumes (PTVs) for the bones, ribs, and spleen were covered by 12 Gy. The dose conformity index, D80, D50, and D10, for PTVs and organs at risk (OARs) for the IMPT plans were quantified and compared with the corresponding VMAT plans.

**Results:**

The mean dose for most of the OARs was reduced substantially (5% and more) in the IMPT plans for TMI in comparison with VMAT plans except for the esophagus and thyroid, which experienced an increase in dose. This dose reduction is due to the fast dose falloff of the distal Bragg peak in the proton plans. The conformity index was found to be similar (0.78 vs 0.75) for the photon and proton plans. IMPT plans provided superior superficial dose coverage for the skull and ribs in comparison with VMAT because of increased entrance dose deposition by the proton beams.

**Conclusion:**

Treatment plans for TMI generated with IMPT were superior to VMAT plans mainly due to a large reduction in the OAR dose. Although the current IMPT-TMI technique is not clinically practical due to the long overall treatment time, this study presents an enticing alternative to conventional TMI with photons by providing superior dose coverage of the targets, increased sparing of the OARs, and enhanced radiobiological effects associated with proton therapy.

## Introduction

Total body irradiation (TBI) is widely used as a conditioning treatment regimen for hematopoietic stem cell transplantation (HCT) (1). Conventional TBI cannot deliver higher radiation dose safely without increasing toxicity to surrounding normal tissues from excess dose especially the lungs, negating any potential advantage to overall survival (2–8). Additionally, the conventional TBI technique is associated with non-uniform dose distributions, high doses to organs at risk (OARs), and hot spots in normal tissues (9). To overcome this obstacle, total marrow irradiation (TMI) with helical tomography was developed, allowing for dose reduction to normal tissues while providing conformal dose coverage to the planning target volumes (PTVs) (9–16). With initial clinical trials demonstrating TMI to be successful for patient treatment (17), the expansion of TMI into advanced clinical modalities could potentially impact the efficiency, quality, and outcomes of patient treatment.

In recent years, volumetric modulated arc therapy (VMAT) for TMI has been used to provide highly conformal dose distributions to the TMI targets and lower doses to OARs, which is superior to conventional radiation treatment techniques (18, 19). Advancement in dose delivery techniques and radiation therapy modalities particularly proton therapy provides an appealing avenue for TMI treatment. Proton therapy provides conformal dose distributions with fast dose falloff and no exit doses due to the Bragg peak, and it is associated with higher radiobiological effective doses compared with photon therapy (20). Currently, no attempts have been made to adapt TMI to a proton therapy because of the clinical and technical limitations. The goal of this study is to investigate treatment planning of TMI using intensity-modulated spot-scanning proton therapy (IMPT). The dosimetric parameters of the intensity-modulated proton plans were evaluated and compared with the corresponding TMI plans generated with VMAT using photon beams.

## Methods

### Patient selection and organ contouring

The computed tomography (CT) images with 512 × 512 pixels with 6-mm slice thickness for five patients who were previously treated with craniospinal irradiation (CSI) were used for TMI treatment planning in this study. **Table 1** lists the demographics of the patients used in this study. Patients were positioned headfirst supine for simulation, with the CT images for these patients consisting of nearly whole-body scans covering from the top of the skull past the pelvis. The clinical target volume (CTV) was defined as all the bones and lymph nodes from the vertex to the mid femur except for the humeri, ulnae, radii, and hands. Standard CSI patient setup required setting the arms away from the body to avoid any extra irradiation to the extremities; thus, the extremities (arms) were excluded from dosimetric calculation and assessment in this study. The bones outlined for CSI were modified such that a custom-made 5-mm margin to the bones with a 1-mm cropping away from OARs except for cranial bones where a 2-mm margin was applied for the PTV. This bone PTV matched with previously reported dosimetric margins used in TMI treatment planning (21). Mandible and maxillary structures were excluded from the bone PTV following the methodology of Wong et al. (11). The same CT images with the outlined TMI targets and OARs were used for both IMPT and VMAT planning in the proton and photon treatment planning systems, respectively.

**Table 1:** Patient demographics used for treatment planning.

TMI study designation	Sex	Age (years)	Length of upper-body PTV (cm)	Weight (kg)	Volume of PTV (cm^3^)
TMI_001	M	9	69	34.3	4,027.6
TMI_002	F	9	72.76	24.8	3,168.9
TMI_003	M	13	84.76	37.6	6,460.7
TMI_004	M	4	59.03	16	2,451.2
TMI_005	F	18	88.13	50	7,534.5

TMI, total marrow irradiation; PTV, planning target volume.

### Intensity-modulated spot-scanning total marrow irradiation planning technique

**Figure 1:**
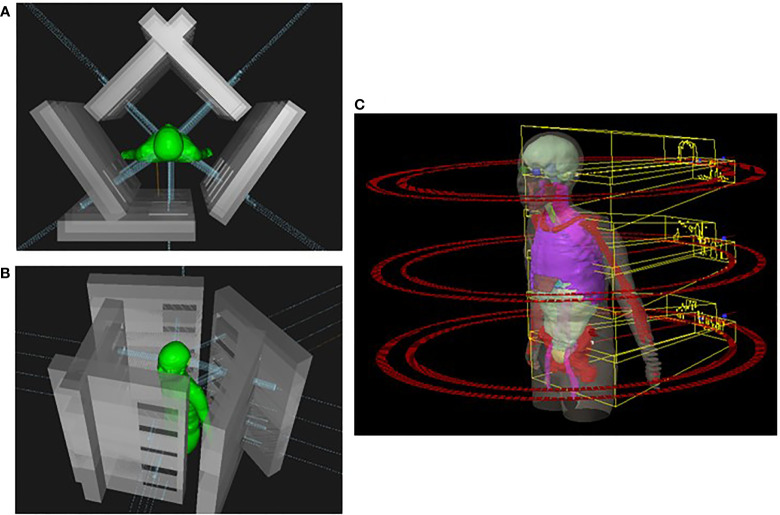
**(A)** Head-to-toe view of IMPT plan taken from Raystation planning system. Five beams were used for each isocenter in this example. **(B)** Side view of IMPT plan. **(C)** Representation of a VMAT-TMI plan with arcs and isocenters from Eclipse planning system. IMPT, intensity-modulated spot-scanning proton therapy; VMAT, volumetric modulated arc therapy; TMI, total marrow irradiation.

Intensity-modulated proton plans for TMI were created using the Monte Carlo dose-calculation algorithm in the Raystation 11A treatment planning system with spot-scanning proton beams from the MEVION S250i Hyperscan proton therapy system (MEVION Medical Systems, Littleton, MA, USA). The proton plans were generated with four to five isocenters placed midline along the cranial–caudal direction of the patient where each isocenter included a set of five beams with a total of 20–25 beams to cover the whole body. Four proton beams were placed at gantry angles of 45° and 125° with table rotations of 0° and 180°, and a fifth beam was directed along the patient posteriorly at a gantry angle of 180°. The field size was set to the maximum of 20 × 20 cm with a 2-cm overlap for each field in the cranial–caudal direction as seen in **Figures 1A, B**. Several different beam configurations were tested, the results of which can be found in the **Supplementary Material**. The dose calculation grid was set to a 2-mm resolution to consider the variations in high-dose gradient regions. Multi-field optimization (MFO) technique was used with the tolerance set to 1E−5 and a maximum number of iterations of 200 to achieve a conformal dose coverage of the different targets. Raystation reports dose in units of cGy-RBE, which includes the enhanced relative biological effectiveness (RBE) with proton beams, allowing for direct comparisons between VMAT-TMI and IMPT-TMI. The parameters of the spot filtering setting used for dose optimization and calculation are given in **Table 2** for the IMPT plans.

**Table 2:** Settings of dose parameters used for proton optimization and planning.

Spot filtering settings			
Iterations before spit filtering		40	
Min spot meterset (MU/fx)		0.135	
Max spot meterset (MU/fx)		42	
Meterset limit margin (%)		5	
Proton plan optimization			
ROI	Description		Weight
PTV_TMI	Min dose 1,100 cGy (RBE)		250
PTV_TMI	Max dose 1,440 cGy (RBE)		1,000
Airway	Max dose 650 cGy (RBE)		100
Bladder	Max dose 650 cGy (RBE)		100
Bowel	Max dose 650 cGy (RBE)		100
Brain	Max dose 650 cGy (RBE)		100
Esophagus	Max dose 650 cGy (RBE)		100
Heart	Max dose 400 cGy (RBE)		100
LT optic nerve	Max dose 650 cGy (RBE)		100
LT orbit	Max dose 650 cGy (RBE)		100
Parotids	Max dose 650 cGy (RBE)		100
RT optic nerve	Max dose 650 cGy (RBE)		100
RT orbit	Max dose 650 cGy (RBE)		100
Spleen	Min dose 1,200 cGy (RBE)		100
Stomach	Max dose 650 cGy (RBE)		100
Thyroid	Max dose 650 cGy (RBE)		100
Total lung	Max dose 650 cGy (RBE)		250
Total kidneys	Max dose 750 cGy (RBE)		100

ROI, region of interest; PTV, planning target volume; TMI, total marrow irradiation; RBE, relative biological effectiveness.

### Volumetric modulated arc therapy–total marrow irradiation planning technique

The VMAT-TMI treatment plans were generated with the Eclipse-V15 treatment planning system with the analytical anisotropic dose-calculation algorithm (AAA) using a Varian Trilogy machine and a Millennium MLC system (Varian Medical Systems, Palo Alto, CA, USA). Photon optimization settings were done using extended convergence mode with a 2-mm dose grid resolution for all the VMAT plans. The beam design with arcs and isocenters for VMAT-TMI is shown in **Figure 1C**. Three to four isocenters were used for treatment planning, which were separated by 24 cm in the cranial–caudal direction. The collimators were rotated by 90° to enable the use of asymmetric jaws, allowing for full-range travel of the MLC for intensity modulation following the previously reported methodology (15, 18, 19, 22). Photon treatment fields use full arc rotations starting from 181° in the clockwise direction and 179° in the counterclockwise direction, with large fields of 30 × 40 cm^2^. A 2-cm overlap in the cranial–caudal direction between adjacent arcs for the different isocenters was planned to ensure appropriate dose deposition in the junction regions.

### Plan comparison and analysis

The different PTVs for the TMI treatment planning consisted of the structures spine, ribs, skull, lymph nodes, and spleen, which were covered with a total dose of 12 Gy, and the OARs included the bladder, esophagus, eyes, heart, kidneys, lungs, optic nerves, parotids, small intestine, stomach, and thyroid, which were constrained to achieve dose sparing within tolerance doses as reported from Aydogan et al. (19). The contours were outlined initially by a medical physicist using intensity-level thresholding and manual contouring tools in the Eclipse treatment planning system and then reviewed and approved by a radiation oncologist. The dose conformity index, D80, D50, and D10, for PTVs and OARs for the IMPT plans were quantified and used for dose evaluation and comparison with the corresponding VMAT plans.

Statistical analysis was performed using GraphPad Prism v 7.04 (GraphPad Software Inc., La Jolla, CA, USA). The outliers were identified using a robust non-linear regression method, ROUT (Q = 1%, ‘Q’ is the maximum desired false discovery rate), which were assessed for the different targets and OARs used in the TMI treatment planning. Multiple group comparisons were performed with a one-way ANOVA test, correcting for multiple comparisons. Group comparisons were performed with an unpaired two-tailed Student’s test. A p-value of ≤.05 was considered statistically significant.

## Results

**Figure 2:**
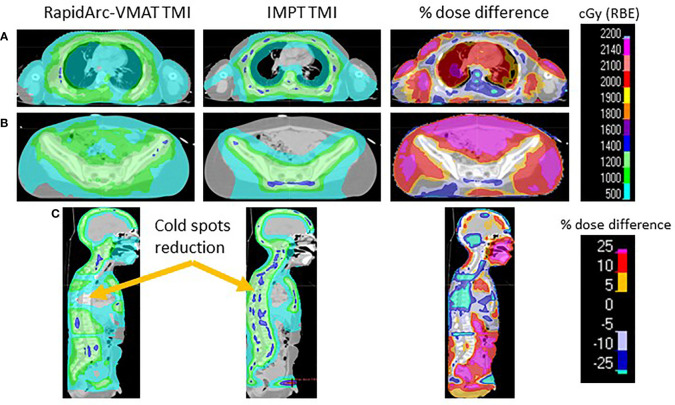
**(A)** Comparing the VMAT, IMPT, and percentage dose difference of the thoracic cavity. **(B)** Pelvis region comparing TMI plans. **(C)** Sagittal view demonstrating the cold spots present in VMAT-TMI. VMAT, volumetric modulated arc therapy; IMPT, intensity-modulated spot-scanning proton therapy; TMI, total marrow irradiation.

The VMAT-TMI dose distributions and the corresponding IMPT dose distributions with the dose difference between the two plans for patient 1 are shown in **Figure 2**. **Figures 2A, B** show the axial dose distributions for the thoracic cavity and pelvis regions, which demonstrated dose reductions in the lung and bowel regions caused by the falloff after the distal Bragg peak of the proton plans in comparison with the corresponding photon plans. Another advantage of the proton plans was the reduction of cold dose spots in the T-spine and L-spine regions by 33% ± 14% from the VMAT plan as seen in **Figure 2C**. This was achieved by heavily weighting the lung OAR during the proton plan optimization.

**Figure 3:**
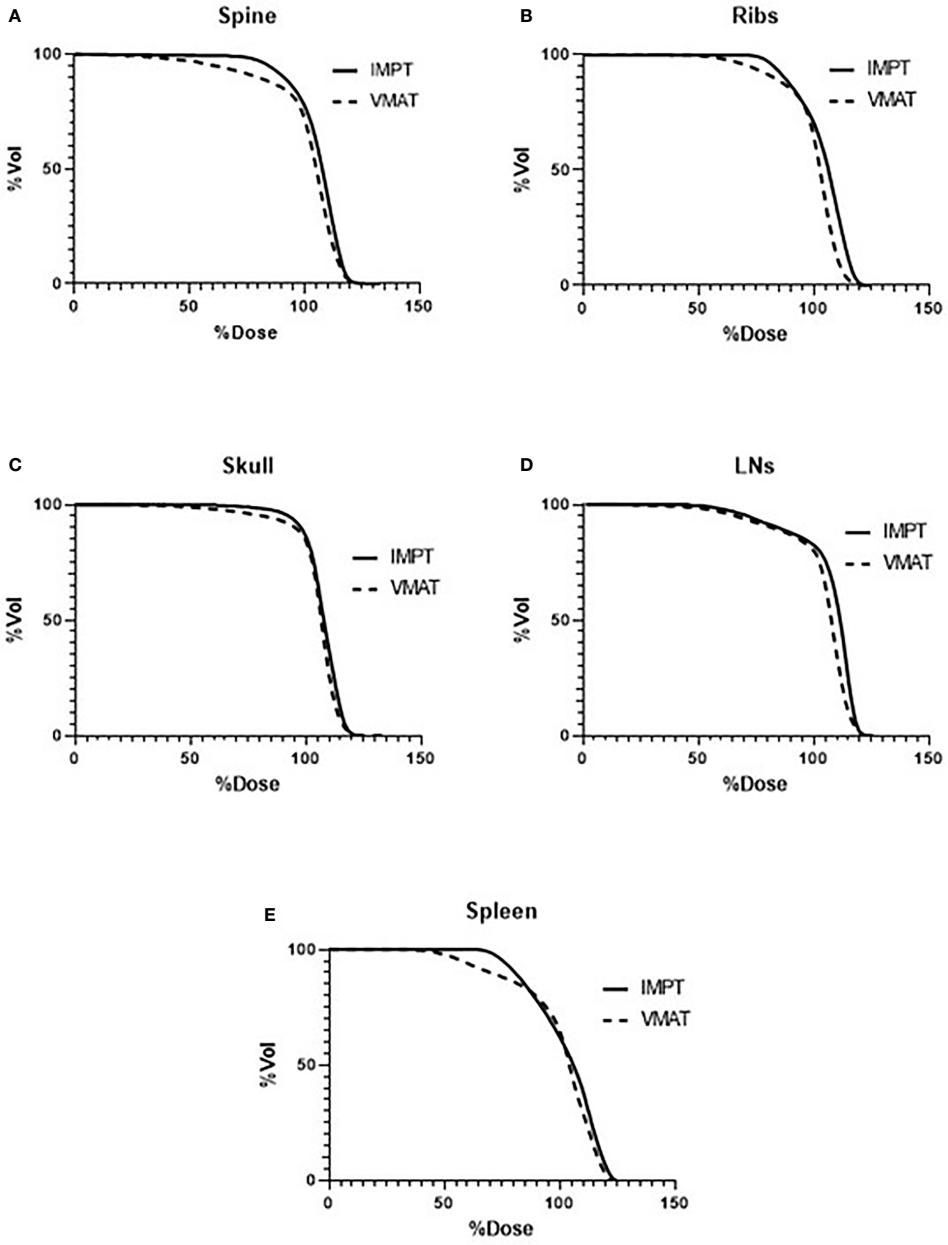
Average DVHs comparing VMAT to IMPT plans of the targets: **(A)** spine, **(B)** ribs, **(C)** skull, **(D)** lymph nodes, and **(E)** spleen. DVHs, dose–volume histograms; VMAT, volumetric modulated arc therapy; IMPT, intensity-modulated spot-scanning proton therapy.

**Figure 4:**
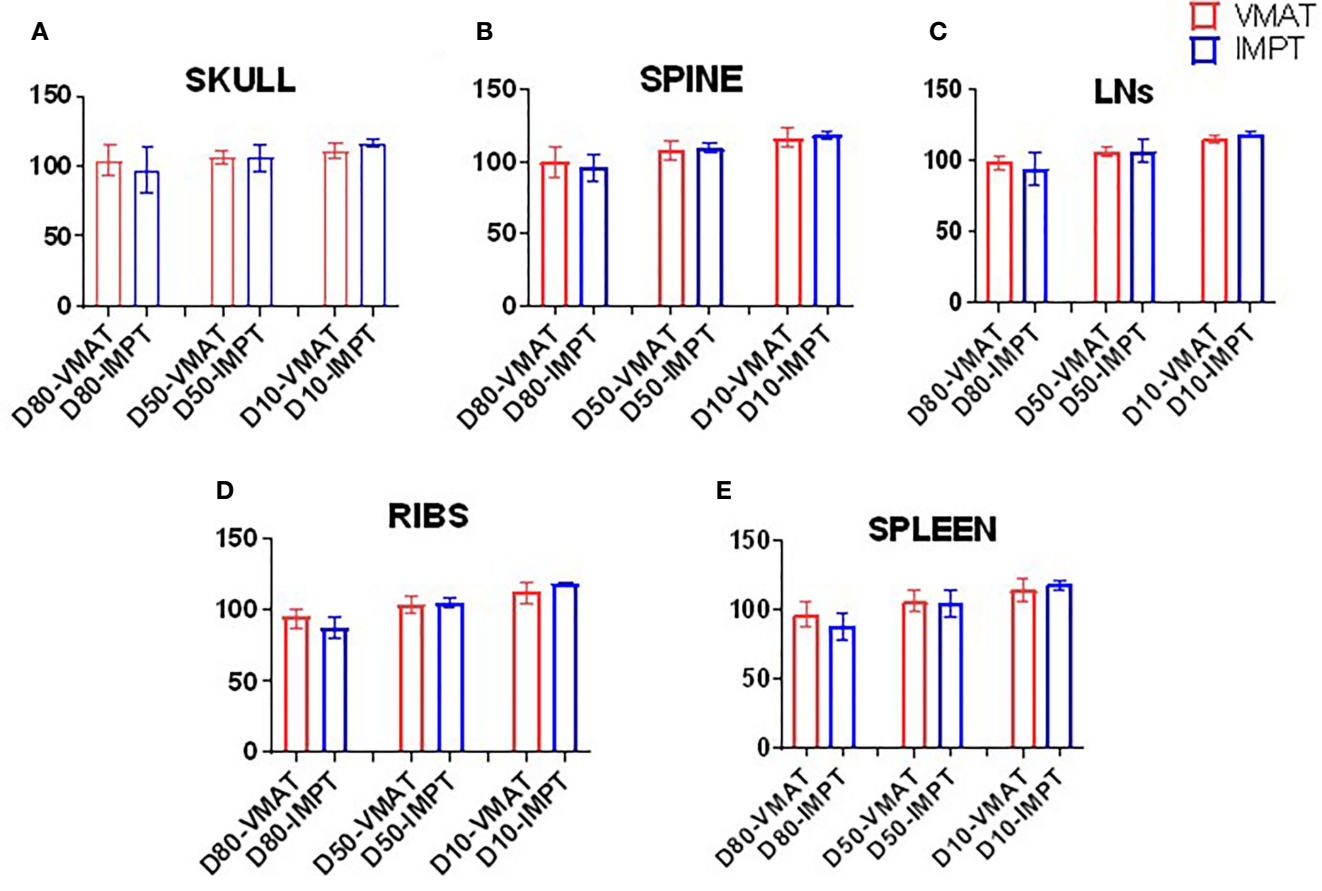
Bar graphs of the D80, D50, and D10 of targets. **(A)** Skull, **(B)** spine, **(C)** lymph nodes, **(D)** ribs, and **(E)** spleen.

**Figure 3** lists the D80, D50, and D10 dosimetric results of both VMAT and IMPT plans for each patient for the PTVs: skull, ribs, spine, spleen, and lymph nodes. **Figure 4** shows the D80, D50, and D10 in bar graph format for each PTV. The D80 coverage for the skull, ribs, and spleen was lower by 7.4%, 7.3%, and 8.7%, respectively, for IMPT plans compared to VMAT. Despite the lower D80 dose in IMPT plans, it was not significantly different when compared to VMAT plans (*p* >.08). The D10 for the skull was 6.3% higher in the IMPT plans compared to VMAT (*p* = .02). The maximum dose was higher in IMPT plans by 16.8% compared to VMAT-TMI (*p* <.05). The dose coverage uncertainty was 5% higher in the D80 compared to D10 for IMPT plans. All the other dose metrics for PTV dose coverages were within ±5% for the proton and photon modalities.

**Figure 5:**
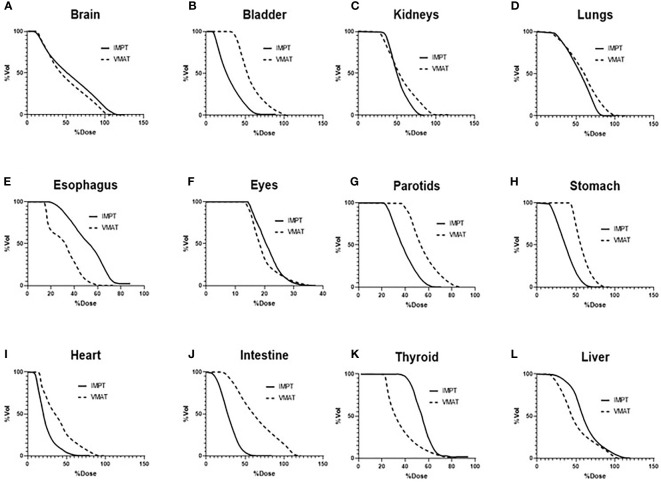
Average DVHs of the OARs comparing VMAT to IMPT plans: **(A)** brain, **(B)** bladder, **(C)** kidneys, **(D)** lungs, **(E)** esophagus, **(F)** eyes, **(G)** parotids, **(H)** stomach, **(I)** heart, **(J)** intestine, **(K)** thyroid, and **(L)** liver. DVHs, dose–volume histograms; OARs, organs at risk; VMAT, volumetric modulated arc therapy; IMPT, intensity-modulated spot-scanning proton therapy.

**Table 3** lists the mean dose for each OAR structure. The OARs that experienced the largest reductions in the mean doses in the IMPT plans were the bladder (50.4%) and intestine (50.4%). The fast dose falloff in proton plans and reduced scatter radiation led to less secondary radiation in these structures as compared to VMAT plans. The mean doses were higher in the esophagus and thyroid by 43.4% and 33.8%, respectively, in the IMPT plans (*p* <.01). **Figure 5** displays the average dose–volume histograms (DVHs) for the OARs, which show the dose sparing for organs such as the stomach, parotids, bladder, and intestine.

**Table 3:** Mean OAR doses and percentage differences between the VMAT and IMPT plans.

Average OAR doses (cGy) for n = 5 patients				
OAR	VMAT-TMI	IMPT	%diff	p-Value
Brain	612.8	609.0	0.6	0.96
Bladder	772.6	383.0	50.4	<0.01
Esophagus	401.0	575.0	−43.4	<0.01
Eyes	293.6	287.8	2.0	0.81
Heart	483.8	418.2	13.6	0.37
Intestine	809.0	401.0	50.4	<0.01
Kidneys	709.4	542.8	23.5	0.01
Lungs	732.6	632.8	13.6	0.02
Parotids	468.4	437.0	6.7	0.57
Stomach	491.8	460.8	6.3	0.72
Thyroid	415.8	556.2	−33.8	<0.01
Liver	630.4	634.8	−0.7	0.92

OAR, organ at risk; VMAT, volumetric modulated arc therapy; TMI, total marrow irradiation; IMPT, intensity-modulated spot-scanning proton therapy.

## Discussion

This is one of the first simulation studies demonstrating the potential of intensity-modulated spot-scanning proton therapy as an alternative technique for total marrow irradiation treatment. The IMPT plans achieved similar dosimetric coverage as compared to VMAT while reducing the dose to OARs such as the lungs and kidneys. In certain PTVs such as the T-spine, the bone PTV experienced a loss in dose coverage due to normal tissue sparing in VMAT; however, IMPT has the advantage of not having such cold spots. This study demonstrated several dosimetric advantages of IMPT over VMAT for TMI, which can provide potential avenues for further TMI treatment planning development and possible clinical implementation.

### Clinical advantages of proton treatments versus photon radiation treatment planning for bone marrow environment

One of the main advantages of protons over photons is reduced dose deposition in normal tissue and the enhanced deposition of radiation dose in the target region set by the spread-out Bragg peak of charged particles. This physical characteristic of the protons is reflected in current dosimetric planning in which the IMPT plans achieved similar target coverage compared to VMAT planning while providing a reduction in the dose deposition to OARs. This dose reduction to OARs in IMPT plans can be used to justify dose-escalation studies, which may provide better disease control as suggested by previous work (23). Based on several *in vitro* studies, the RBE of the protons is considered 1.1, which is superior to that of photons (24). Furthermore, there may be increased RBE due to enhanced linear energy transfer when protons are close to the Bragg peak (25). A recent study by Zuro et al. (26) suggests that a certain level of radiation dose to the body may be essential for sustained donor marrow engraftment, indicating that a complex biological mechanism controls donor cell homing and expansion. Therefore, beyond toxicity reduction, the mechanism of how proton TMI will support successful donor cell engraftment requires further investigation. Furthermore, a better understanding of the RBE of protons in the context of leukemia cell killing and the effects of proton therapy on the bone marrow microenvironment in the *in vivo* system will strengthen the clinical translation of this technique.

### Potential impact of proton arc therapy on total marrow irradiation

Photon TMI was originally conceived with arc therapy using the TomoTherapy system and later the RapidArc system from Varian. Several works have demonstrated the benefits of VMAT versus conventional photon treatment planning for a variety of different sites (27–29). Typically, VMAT offers superior dose coverage and reduction of OAR dose as compared to step-and-shoot intensity-modulated radiation therapy (IMRT) at the cost of increased planning time. Currently, proton arc therapy does not exist for clinical use; however, recent technological advancements have demonstrated potential clinical feasibility (30, 31). Several proton arc studies have even shown superior dose coverage of the tumor and OAR sparing, which could prove to be potentially superior dosimetrically to VMAT-TMI (32). Another potential benefit of proton arc therapy is a reduction in clinical treatment times (32). Currently, the Raystation system allows for fast optimization, usually with 20–30 min of total planning time for proton plans compared to Eclipse arc planning, which can be 3–6 h for photon plans. However, the proton dose delivery time for regular proton fields directed from several discrete angles is estimated to be more than 2 h due to the nature of spot-scanning proton therapy, as in this simulation study. Proton arc beams can greatly reduce the total treatment time required for dose delivery.

## Conclusion

This simulation study demonstrates several dosimetric advantages of proton therapy versus photon therapy for TMI. IMPT plans displayed better dose conformity, reduction in cold dose spots inside the PTVs, and reduced OAR doses as compared to VMAT-TMI except for the esophagus and thyroid. Technical advancement in the treatment planning and dose delivery of proton therapy such as the development of arc-based proton therapy might enable the feasibility and clinical implementation of proton therapy for TMI. In addition to these dosimetric advantages, proton therapy may have superior radiobiological effects for the TMI treatment, which requires further investigation.

## Data Availability Statement

The original contributions presented in the study are included in the article/**supplementary material**. Further inquiries can be directed to the corresponding author.

## Author Contributions

DZ: First and Corresponding author; GV: first author; JC: first author; YC: second author; CHa: second author; CHe: second author; SA: second author; SH: senior author; IA: senior author. All authors contributed to the article and approved the submitted version.

## Conflict of Interest

The authors declare that the research was conducted in the absence of any commercial or financial relationships that could be construed as a potential conflict of interest.

## Publisher’s Note

All claims expressed in this article are solely those of the authors and do not necessarily represent those of their affiliated organizations, or those of the publisher, the editors and the reviewers. Any product that may be evaluated in this article, or claim that may be made by its manufacturer, is not guaranteed or endorsed by the publisher.
