# Supplementary material for: Treatment planning of total marrow irradiation with intensity-modulated spot-scanning proton therapy
Source: Front Oncol. 2022 Jul 28;12:955004. doi: 10.3389/fonc.2022.955004 (PMC9365973; doi:10.3389/fonc.2022.955004)
Supplement: Supplementary file 1 [file DataSheet_1.docx]

Supplement Section

To assess if the 5 field beam arrangement performed the best dosimetrically for TMI a test cases were created using alternative beam arrangement based on current methodology for CSI treatments. A single posterior beam was placed along the patient’s spine. Supplement Figure 1a displays the layout for a single posterior TMI beam plan with Figure 1b displaying the DVH. The single posterior beam plan had the D80 for the PTV (30%), skull (20%), and ribs (23%) reduced compared to 5 field TMI. Additionally, the brain and heart mean dose increased by 13% and 8% respectively.
