# Supplementary figures and images for: Treatment planning of total marrow irradiation with intensity-modulated spot-scanning proton therapy
Source: Front Oncol. 2022 Jul 28;12:955004. doi: 10.3389/fonc.2022.955004 (PMC9365973; doi:10.3389/fonc.2022.955004)

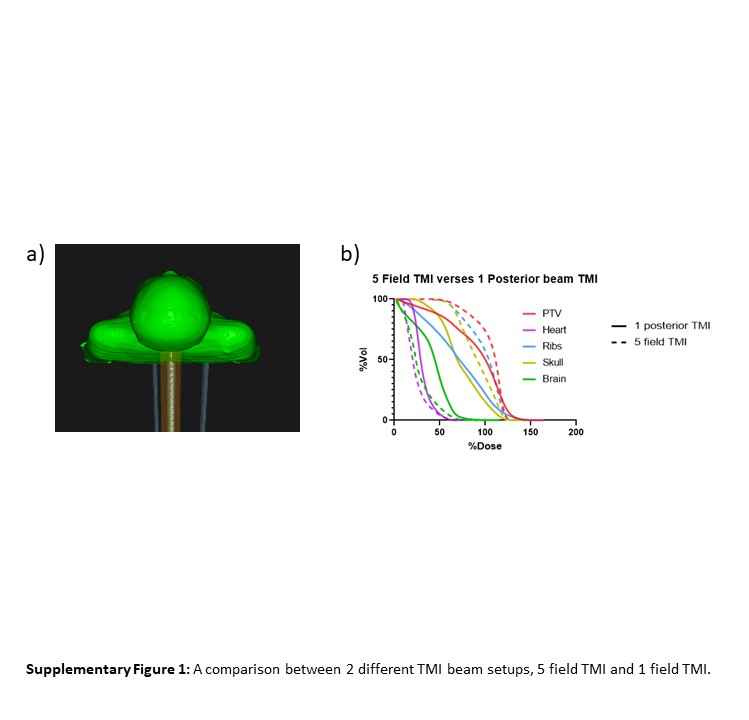

Supplement: Supplementary file 2 [file Image_1.jpeg]
